# Association of awake prone positioning with clinical outcomes in severe immune checkpoint inhibitor-associated pneumonitis with acute hypoxemic respiratory failure: a propensity-matched retrospective cohort study

**DOI:** 10.3389/fmed.2026.1880206

**Published:** 2026-07-31

**Authors:** Changhao Yin, Yan Qiang, Zhen Mei, Changzhen Qi, Qian Chen, Jisheng Zhou, Xiaoyan Fan

**Affiliations:** Intensive Care Unit (ICU), Jiangsu Cancer Hospital, Nanjing, Jiangsu Province, China

**Keywords:** acute hypoxemic respiratory failure, awake prone positioning, critical care, immune checkpoint inhibitor-associated pneumonitis, propensity score matching

## Abstract

**Objective:**

Severe immune checkpoint inhibitor (ICI)-associated pneumonitis can lead to acute hypoxemic respiratory failure and substantial mortality. This study evaluated the association between awake prone positioning (APP) and clinical outcomes in patients with clinically diagnosed severe ICI-associated pneumonitis.

**Methods:**

In this single-center retrospective cohort study, 120 propensity-matched patients with clinically diagnosed severe ICI-associated pneumonitis and acute hypoxemic respiratory failure between January 2019 and September 2024 were analyzed. Infectious pneumonia, COVID-19 pneumonia, opportunistic infection, tumor progression, radiation pneumonitis, cardiogenic pulmonary edema, pulmonary embolism, diffuse alveolar hemorrhage, and other dominant alternative causes of respiratory failure were excluded through structured diagnostic adjudication. Patients were classified into the APP group (*n* = 60, cumulative APP duration ≥10 h during the first ICU week) or control group (*n* = 60) according to documented positioning exposure. Primary outcomes were dynamic PaO₂/FiO₂ ratio trajectories and endotracheal intubation rates; secondary outcomes included ICU length of stay and 28-day all-cause mortality. Sensitivity analyses addressed pandemic period, APP exposure timing, FiO₂ documentation, and competing risk for ICU discharge.

**Results:**

APP was associated with a higher PaO₂/FiO₂ ratio at 168 h (268 ± 52 vs. 201 ± 49 mmHg, *p* < 0.001) and a lower intubation rate [11/60 (18.3%) vs. 27/60 (45.0%), *p* = 0.001; adjusted OR = 0.24, *p* = 0.002]. ICU stay was shorter in the APP group (8.5 vs. 12.3 days, *p* < 0.001). The APP group also had lower observed 28-day mortality than the control group [11/60 (18.3%) vs. 21/60 (35.0%), *p* = 0.039]. Findings were directionally consistent in sensitivity analyses, although effect estimates were attenuated in APP initiation-based and landmark analyses. Protocol-defined adverse events were comparable between groups.

**Conclusion:**

In this retrospective propensity-matched cohort study, APP was associated with improved oxygenation and lower intubation rates in patients with clinically diagnosed severe ICI-associated pneumonitis. Associations with shorter ICU stay and lower observed 28-day mortality should be interpreted cautiously because of the observational design, potential residual confounding, and possible survivor or tolerance bias.

## Introduction

Immune checkpoint inhibitors (ICIs) have become integral components of anticancer therapy, but immune-related pulmonary toxicity remains a clinically important complication. ICI-associated pneumonitis is an inflammatory lung injury that may present with dyspnea, cough, new pulmonary infiltrates, and acute hypoxemic respiratory failure. Because its clinical and radiologic manifestations may overlap with infectious pneumonia, tumor progression, radiation pneumonitis, and other causes of respiratory deterioration, the diagnosis requires integration of ICI exposure history, chest imaging, clinical course, and exclusion of alternative dominant causes, particularly microbiologically confirmed pulmonary infection (1–3).

Severe ICI-associated pneumonitis may progress to acute hypoxemic respiratory failure, for which intubation and invasive mechanical ventilation are sometimes required when noninvasive respiratory support fails. However, the invasiveness of these interventions and the associated risks, including ventilator-associated pneumonia (VAP), remain clinically important concerns. In mechanically ventilated ICU patients, VAP has been reported at a high incidence, particularly among patients receiving orotracheal intubation, and multidrug-resistant pathogens are frequently involved (4). These risks provide a rationale for exploring adjunctive noninvasive strategies that may improve oxygenation and reduce the need for endotracheal intubation when clinically appropriate. One such strategy is awake prone positioning (APP), in which patients are placed in a prone or semi-prone position while remaining conscious and breathing spontaneously. APP may improve oxygenation by enhancing ventilation-perfusion matching and recruitment of dependent lung regions (5).

The potential benefits of APP have been widely studied in COVID-19-related acute hypoxemic respiratory failure, where it has been associated with improved oxygenation and reduced intubation risk (5, 6). However, its application in patients with severe ICI-associated pneumonitis is not well established. This population differs from patients with infectious pneumonia because respiratory failure may be driven primarily by immune-mediated alveolar and interstitial inflammation rather than microbiologically confirmed infection, and treatment frequently involves corticosteroids and complex oncologic decision-making. Given the increasing use of ICIs in cancer treatment and the clinical importance of immune-related pulmonary toxicity, it is necessary to evaluate whether APP is associated with improved respiratory and clinical outcomes in patients with severe ICI-associated pneumonitis.

This study aimed to evaluate the association between APP and clinical outcomes in patients with clinically diagnosed severe ICI-associated pneumonitis and acute hypoxemic respiratory failure after exclusion of microbiologically confirmed infectious pneumonia and other dominant alternative causes of respiratory failure. Specifically, we assessed dynamic PaO₂/FiO₂ ratio trajectories, endotracheal intubation rates, intensive care unit (ICU) length of stay, 28-day all-cause mortality, and protocol-defined adverse events. By focusing on this clinically distinct population, the study sought to provide real-world evidence on the feasibility and potential clinical role of APP as an adjunctive respiratory support strategy for severe ICI-associated pneumonitis.

## Methods

### Study design

This single-center retrospective cohort study, conducted in accordance with the STROBE (Strengthening the Reporting of Observational Studies in Epidemiology) guidelines, evaluated the association between APP and respiratory outcomes in adults with clinically diagnosed severe ICI-associated pneumonitis and acute hypoxemic respiratory failure. Electronic medical records (EMR) from a hospital in Jiangsu Province were systematically reviewed for patients admitted between January 1, 2019, and September 30, 2024. To ensure data fidelity, a random subset of 10% of cases underwent independent dual verification by trained research coordinators, with discrepancies resolved through adjudication by a critical care physician. The study protocol received ethical approval from the Institutional Review Board of Jiangsu Cancer Hospital (approval number: 2023-002). A waiver of informed consent was granted as the research involved anonymized retrospective data analysis without intervention, adhering to local regulations and the Declaration of Helsinki. All data were handled confidentially with strict adherence to privacy protection protocols.

### Study population and grouping

The initial source population was identified through systematic query of the institutional Critical Care Database using structured EMR filters, including International Classification of Diseases, Tenth Revision (ICD-10) code J18.9, which was used only as a broad screening code for pulmonary infiltrates or pneumonia-like clinical documentation, and T45.1X5A, which captured adverse effects related to antineoplastic or immunomodulatory therapy, combined with Current Procedural Terminology (CPT) codes 94,002–94,005 for oxygen therapy management. Between January 1, 2019, and September 30, 2024, all adult ICU admissions meeting the initial screening criteria underwent three-stage validation: automated flagging by the hospital clinical data warehouse, manual chart review by two independent critical care physicians using REDCap electronic case report forms, and final adjudication by a senior intensivist for diagnostic uncertainty. Final eligibility was based on clinically diagnosed severe ICI-associated pneumonitis rather than ICD codes alone. Discrepancies were resolved through consensus discussion.

Because immune checkpoint inhibitor (ICI)-associated pneumonitis is a clinical diagnosis of exclusion, final case identification was based on a predefined diagnostic adjudication process rather than administrative codes alone. Severe ICI-associated pneumonitis was diagnosed when all of the following conditions were met: (1) current or recent ICI exposure within 90 days before respiratory deterioration; (2) new or worsening respiratory symptoms, including dyspnea, cough, increased oxygen requirement, or acute hypoxemic respiratory failure; (3) new or progressive pulmonary infiltrates on chest computed tomography or chest radiography judged compatible with ICI-associated pneumonitis, including organizing pneumonia-like, ground-glass, interstitial, or diffuse inflammatory patterns when documented; (4) PaO₂/FiO₂ ratio <300 mmHg with oxygen requirement ≥5 L/min; and (5) no microbiologically confirmed infectious pneumonia, opportunistic pulmonary infection, SARS-CoV-2 infection or COVID-19 pneumonia considered the dominant cause of respiratory failure, tumor progression, radiation pneumonitis, cardiogenic pulmonary edema, pulmonary embolism, diffuse alveolar hemorrhage, or other dominant alternative cause of respiratory failure after structured chart review and physician adjudication (1–3).

The diagnostic adjudication process consisted of independent review by two critical care physicians using standardized electronic case report forms, followed by final review by a senior intensivist when diagnostic uncertainty or disagreement was present. Available diagnostic evidence included the temporal relationship between ICI exposure and respiratory deterioration, chest imaging reports, arterial blood gas results, inflammatory markers, microbiological results from sputum or bronchoalveolar lavage when available, SARS-CoV-2 nucleic acid or antigen testing when available, respiratory viral testing when clinically performed, fungal or opportunistic infection testing when clinically indicated, bronchoscopy findings, antibiotic response, corticosteroid response, oncologic imaging, echocardiographic or volume-status assessment when available, coagulation and hemoptysis-related documentation, clinician-documented COVID-19 diagnosis, and multidisciplinary clinical notes. Cases were excluded when microbiologically confirmed infectious pneumonia, opportunistic pulmonary infection, COVID-19 pneumonia, tumor progression, radiation pneumonitis, cardiogenic pulmonary edema, pulmonary embolism, diffuse alveolar hemorrhage, or another non-ICI cause was considered the dominant explanation for respiratory failure. Therefore, the study population should be interpreted as patients with clinically diagnosed severe ICI-associated pneumonitis and acute hypoxemic respiratory failure, rather than patients with infectious pneumonia, COVID-19 pneumonia, or mixed pulmonary infiltrates of uncertain dominant etiology.

Inclusion criteria required all the following: (1) age ≥18 years with histologically confirmed solid or hematological malignancy; (2) current or recent ICI therapy within 90 days before respiratory deterioration, including anti-programmed death-1 (anti-PD-1), anti-programmed death ligand-1 (anti-PD-L1), or anti-cytotoxic T-lymphocyte-associated protein 4 (anti-CTLA-4) agents; (3) clinically diagnosed severe ICI-associated pneumonitis with acute hypoxemic respiratory failure according to the diagnostic adjudication process described above (1–3); and (4) complete electronic medical record (EMR) documentation, including arterial blood gas (ABG) results, nursing flow sheets, respiratory therapy records, and chest imaging reports.

Exclusion criteria encompassed any of the following: (1) pre-ICU intubation or tracheostomy; (2) microbiologically confirmed infectious pneumonia or opportunistic pulmonary infection, including positive bacterial or fungal culture from bronchoalveolar lavage (BAL) or quantitative sputum culture meeting institutional microbiological thresholds, positive respiratory viral testing judged clinically causal, documented opportunistic infection, or SARS-CoV-2 infection/COVID-19 pneumonia considered the dominant cause of acute hypoxemic respiratory failure; (3) hemodynamic instability requiring norepinephrine ≥0.5 μg/kg/min or an equivalent vasopressor dose; and (4) absolute contraindications to prone positioning, including unstable spinal fractures, intra-abdominal pressure >20 mmHg, or recent abdominal surgery within 72 h, according to prone-positioning safety recommendations (7).

Patients with clinically diagnosed severe ICI-associated pneumonitis were categorized into two groups based on documented positioning therapy. The awake prone positioning (APP) group received APP exposure confirmed by nursing or respiratory therapy records, with a minimum duration of 2 h per session and cumulative duration ≥10 h during the first ICU week, delivered within the institutional ICU positioning framework, including head-of-bed elevation ≥30° and arm positioning in the swimmer’s posture. The control group received no prone positioning exposure during the ICU stay, with non-prone positions, including supine, lateral, or semi-recumbent positions, permitted but documented as predominant for more than 90% of recorded time according to nursing flow sheets.

### Rationale, nursing management, documentation, and adherence assessment for APP

APP was not assigned randomly but was initiated according to treating-clinician judgment within an institutional ICU positioning framework. This framework specified minimum safety requirements for APP initiation, contraindications, monitoring, nursing management, and documentation, but it did not mandate a fixed daily APP duration, number of sessions, or positioning schedule for all patients. In general, APP was considered for patients with clinically diagnosed severe ICI-associated pneumonitis who remained non-intubated, awake, cooperative, and clinically stable but had persistent hypoxemia despite conventional oxygen therapy, high-flow nasal cannula (HFNC), or non-invasive ventilation (NIV). The rationale for APP initiation was to improve dependent lung recruitment, reduce ventilation-perfusion mismatch, and avoid or delay endotracheal intubation in selected patients with acute hypoxemic respiratory failure.

According to this institutional framework, APP could be initiated when the following minimum criteria were met: PaO₂/FiO₂ ratio <300 mmHg or oxygen requirement ≥5 L/min after initial stabilization, preserved consciousness and ability to cooperate with positioning instructions, ability to protect the airway without active vomiting or high aspiration risk, hemodynamic stability without high-dose vasopressor support, absence of immediate need for endotracheal intubation, and absence of absolute contraindications to prone positioning. Patients did not receive APP when feasibility-limiting factors were documented, including impaired consciousness, delirium or agitation, inability to cooperate, high aspiration risk or active vomiting, positional intolerance, hemodynamic instability, clinician assessment that immediate intubation was required or likely, patient refusal, competing procedures or imaging requirements, nursing workload, staffing availability, or inability to provide continuous monitoring during prolonged positioning.

During APP sessions, oxygen saturation, respiratory rate, heart rate, blood pressure, respiratory support settings, subjective dyspnea, comfort, and tolerance were monitored by ICU nurses and respiratory therapists. Nursing management for intolerance included reassurance, explanation of the expected duration and purpose of positioning, adjustment of arm and head position, use of pillows or foam supports under the chest, pelvis, knees, and pressure-prone areas, gradual transition from lateral to semi-prone and then prone positioning when needed, and temporary reduction of session intensity or duration according to patient tolerance. Analgesics, antiemetics, anxiolytic measures without excessive sedation, and respiratory support adjustments were used according to physician orders and bedside assessment. For positional dyspnea, nurses and respiratory therapists reassessed cannula or mask position, oxygen flow or FiO₂ settings, patient posture, secretion burden, and work of breathing. APP was interrupted when dyspnea worsened despite position adjustment or when the patient could no longer tolerate the position safely.

For transient hypotension or hemodynamic fluctuation during APP, patients were returned to a lateral or supine position, blood pressure and heart rate were reassessed, and fluid status or vasopressor requirements were reviewed by the treating team. APP was resumed only after hemodynamic parameters returned to the pre-session range or were judged clinically stable by the bedside clinician. Pressure injury prevention was performed as part of routine APP nursing care. Preventive materials included foam pads, soft pillows, gel pads when available, and prophylactic protective dressings applied to pressure-prone areas according to bedside nursing assessment. Commonly protected sites included the forehead or cheek, chin, shoulders, anterior chest, iliac crests, knees, and areas compressed by oxygen interfaces, tubing, electrocardiographic leads, or monitoring devices. Nursing measures included skin assessment before APP initiation and after return to a non-prone position, adjustment of head and limb position, swimmer’s-position arm alternation during prolonged sessions, scheduled micro-positioning or limb repositioning, avoidance of direct device-related pressure, and prompt correction of skin redness, pain, numbness, or excessive localized pressure. These preventive materials and nursing measures were documented in nursing flow sheets when performed.

APP was interrupted or discontinued when patients developed sustained oxygen desaturation, worsening respiratory distress, new or worsening hemodynamic instability, vomiting, aspiration concern, pressure-related skin compromise, device displacement, procedure or imaging requirements, or intolerance despite comfort measures and position adjustment. Resumption of APP was considered when the precipitating event resolved, oxygenation and hemodynamics returned to a clinically acceptable range, the patient remained awake and cooperative, airway protection was preserved, and bedside nursing or respiratory therapy staff judged that monitoring could be safely continued. Interrupted sessions that were resumed were recorded as the same APP session when the interruption was brief and the planned session continued; otherwise, resumed positioning was recorded as a new session according to nursing documentation.

APP exposure and adherence were reconstructed from routine ICU nursing flow sheets, respiratory therapy records, and physician progress notes. For each documented APP session, trained abstractors recorded the start time, end time, session duration, positioning angle or head-of-bed elevation when available, concurrent respiratory support mode, interruption or discontinuation, reason for interruption, tolerance-management measures, pressure-injury prevention measures when documented, and whether the session was resumed. Cumulative APP duration during the first ICU week was calculated as the sum of all APP sessions with verifiable start and end times. Protocol-adherent APP was defined as a documented prone or semi-prone session lasting ≥2 h, cumulative APP duration ≥10 h during the first ICU week, and documented positioning angle or head-of-bed elevation ≥30° when angle data were available. Interruptions were defined as premature termination of a planned APP session or temporary return to a non-prone position before the scheduled session endpoint.

Incomplete APP documentation was handled conservatively. Sessions with verifiable start and end times were included in cumulative duration calculations. Sessions with only narrative mention of APP, missing start or end time, or unclear duration were not counted toward cumulative APP duration and were classified as non-adherent in adherence analyses. When APP was clearly documented but positioning angle was missing, the session was retained for exposure classification but was not classified as angle-adherent. Patients were assigned to the APP group only when cumulative APP exposure ≥10 h during the first ICU week could be confirmed from nursing or respiratory therapy records. Patients with insufficient documentation to confirm either APP exposure or predominant non-prone management were excluded before propensity score matching.

Because classification into the APP group required accumulation of ≥10 h of APP during the first ICU week, immortal-time and survivor bias were considered methodologically relevant. The interval from ICU admission to the first documented APP session was therefore calculated for all APP patients. Additional sensitivity analyses were conducted using APP initiation as an alternative exposure definition, regardless of cumulative APP duration, and using 24-h and 48-h landmark populations that excluded patients who were intubated or died before the corresponding landmark time point. These analyses were used to evaluate whether the main findings were driven by survival long enough to accumulate APP exposure.

Because APP feasibility depended on patient cooperation, consciousness level, hemodynamic stability, tolerance, clinician judgment, and bedside nursing resources, confounding by indication and selection bias were anticipated. Propensity score matching (PSM) at a 1:1 ratio was therefore performed using the nearest-neighbor algorithm with a caliper width of 0.2 SD of the logit of the propensity score. Baseline covariates incorporated into the propensity score model included age, Sequential Organ Failure Assessment (SOFA) score, malignancy type (solid vs. hematologic), initial PaO₂/FiO₂ ratio, ICI class, time from last ICI infusion to pneumonitis onset, baseline respiratory support mode (HFNC vs. NIV), vasopressor dependence, and peak methylprednisolone-equivalent glucocorticoid dose within the first 72 h after ICU admission. Nevertheless, unmeasured factors such as patient cooperation, clinician preference, nursing workload, staffing patterns, and bedside tolerance assessment could not be fully captured in the retrospective dataset.

### Variable definitions and data collection

The primary outcomes were evaluated through two complementary analyses: (1) dynamic oxygenation trajectory, defined as serial PaO₂/FiO₂ ratios measured at 24, 48, 72, and 168 h after ICU admission, with values extracted from ABG reports timestamped within ±1 h of the target intervals; and (2) endotracheal intubation rate, defined as the requirement for invasive mechanical ventilation via an endotracheal tube during the ICU stay. Intubation events were adjudicated through structured review of physician notes, respiratory therapy records, ventilator orders, and ABG results. Emergent intubations occurring within 2 h after ICU admission were excluded from the primary intubation endpoint to reduce misclassification of patients who already required immediate invasive ventilation at presentation.

Endotracheal intubation was performed according to the institutional ICU protocol for acute hypoxemic respiratory failure. Although the final decision was made by the treating intensivist, intubation was generally indicated when patients showed persistent clinical deterioration despite optimized non-invasive respiratory support, including conventional oxygen therapy, HFNC, NIV, or APP. Prespecified clinical triggers included persistent or worsening hypoxemia, defined as SpO₂ < 90% or PaO₂/FiO₂ ratio <100 mmHg despite high-level oxygen support; severe tachypnea, defined as respiratory rate >35 breaths/min or rapidly increasing respiratory rate; increased work of breathing or signs of respiratory exhaustion; altered mental status or inability to protect the airway; respiratory acidosis, defined as pH < 7.30 with rising PaCO₂; hemodynamic instability requiring escalating vasopressor support; high aspiration risk or inability to clear airway secretions; or clinician judgment of impending respiratory failure.

For the APP group, paired pre-session and post-session ABG values were collected when available, defined as ABG measurement within 1 h before APP initiation and within 2 h after APP session completion. The immediate physiological response was calculated as the percentage change in PaO₂/FiO₂ ratio: [(post-session PaO₂/FiO₂ ratio − pre-session PaO₂/FiO₂ ratio)/pre-session PaO₂/FiO₂ ratio] × 100%.

Secondary endpoints encompassed three domains: (1) ICU length of stay, calculated from ICU admission to the ICU discharge order in hours, with discharge criteria requiring hemodynamic stability, defined as norepinephrine <0.1 μg/kg/min, oxygen requirement ≤4 L/min via nasal cannula, and physician-documented readiness for transfer; (2) 28-day all-cause mortality after ICU admission, determined through hospital records supplemented by regional death registry linkage; and (3) adverse event profile, including protocol-defined complications: pressure injuries, defined as Stage ≥2 according to the National Pressure Injury Advisory Panel classification (8), clinically significant vomiting, defined as ≥2 episodes requiring antiemetic intervention within 24 h, and aspiration events, defined as radiographic evidence of new infiltrates plus documented witnessed regurgitation in nursing notes.

Comprehensive variables were systematically abstracted from structured electronic sources using Research Electronic Data Capture (REDCap; Vanderbilt University, Version 13.5.0). Extracted variables included baseline characteristics (age, sex, malignancy type and stage, body mass index), immunotherapy parameters (ICI drug name, cumulative cycles, and date of last infusion), disease severity metrics (SOFA score at ICU admission (9), baseline PaO₂/FiO₂ ratio, and vasopressor dependence), and potential confounding interventions, including peak methylprednisolone-equivalent glucocorticoid dose, broad-spectrum antibiotic administration, defined as coverage for Pseudomonas or methicillin-resistant *Staphylococcus aureus*, bronchoscopy utilization, and baseline respiratory support mode.

COVID-19 and temporal practice variables included calendar year of ICU admission, pandemic period, SARS-CoV-2 nucleic acid or antigen test result when available, respiratory viral testing when clinically performed, and clinician-documented COVID-19 pneumonia as a dominant diagnosis. Pandemic period was categorized as pre-pandemic (January 1, 2019 to December 31, 2019), pandemic (January 1, 2020 to December 31, 2022), and post-pandemic (January 1, 2023 to September 30, 2024). SARS-CoV-2 infection or COVID-19 pneumonia judged to be the dominant cause of acute hypoxemic respiratory failure was treated as an exclusion criterion rather than as part of the eligible ICI-associated pneumonitis cohort.

APP-specific covariates included session frequency, daily duration, cumulative duration during the first ICU week, time from ICU admission to the first documented APP session, APP initiation within 24 h and within 48 h after ICU admission, positioning angle or head-of-bed elevation documented in nursing flow sheets, concurrent respiratory support mode, preventive materials or pressure-relief measures when documented, interruption or discontinuation, reason for interruption, whether the session was resumed, and early outcome events before APP eligibility thresholds were reached. Early outcome events included intubation or death before the 24-h landmark, before the 48-h landmark, and before accumulation of 10 h of documented APP exposure.

PaO₂/FiO₂ ratio calculations used FiO₂ values obtained from respiratory device settings recorded in respiratory therapy charts. For HFNC and NIV, FiO₂ was preferentially extracted from device or ventilator settings documented at the time closest to ABG measurement. For HFNC patients, the primary analysis used device-set FiO₂ rather than estimated FiO₂. Each ABG-FiO₂ pair was classified as having complete FiO₂ documentation when device-set FiO₂ was recorded within the prespecified ABG time window. ABG time points without verifiable device-set FiO₂ were not used for PaO₂/FiO₂ ratio calculation. A sensitivity analysis restricted the PaO₂/FiO₂ trajectory model to ABG time points with complete device-set FiO₂ documentation.

To address retrospective data limitations, a four-phase validation framework was implemented: (1) automated logic checks for temporal consistency, such as ICU discharge date ≥ admission date; (2) source verification of 20% randomly selected ABG values against primary laboratory databases; (3) independent dual abstraction for critical endpoints, including intubation events and mortality status, with inter-rater reliability targets of *κ* > 0.85 for categorical variables and intraclass correlation coefficient >0.90 for continuous measures; and (4) endpoint adjudication committee review by an intensivist and an oncology nurse specialist for discordant cases.

### Statistical analysis

Categorical variables were compared using χ^2^ tests or Fisher’s exact tests, as appropriate, whereas continuous variables were analyzed using independent t-tests or Mann–Whitney U tests according to distributional characteristics. For primary outcomes, dynamic PaO₂/FiO₂ ratio trajectories were analyzed using linear mixed-effects models with random patient intercepts, adjusted for respiratory support mode and prone positioning angle. Endotracheal intubation rates were first evaluated using univariate χ^2^ tests, followed by multivariable logistic regression adjusting for age, SOFA score, ICI class, peak methylprednisolone-equivalent glucocorticoid dose, baseline PaO₂/FiO₂ ratio ≤200 mmHg, respiratory support mode, and vasopressor dependence. Twenty-eight-day all-cause mortality was assessed using multivariable logistic regression with the same prespecified covariates as the intubation model.

For ICU length of stay, time to ICU discharge was analyzed using Kaplan–Meier curves with log-rank tests and Cox proportional hazards models stratified by baseline PaO₂/FiO₂ ratio. ICU discharge order was treated as the event in the primary Cox model. Death before ICU discharge was censored in the primary time-to-discharge analysis and was subsequently evaluated as an informative competing event in a Fine-Gray competing-risk sensitivity analysis. Transfer to another ICU was censored at the time of transfer because subsequent ICU discharge status could not be consistently verified from the source records.

Subgroup and exploratory analyses included stratification by baseline PaO₂/FiO₂ ratio (≤200 mmHg vs. >200 mmHg), restriction to protocol-adherent APP, defined as documented positioning angle or head-of-bed elevation ≥30° with ≥2 h per session and cumulative APP duration ≥10 h during the first ICU week, and stratification by respiratory support mode (HFNC vs. NIV). Exploratory duration-response analyses assessed the association between cumulative APP duration and clinical outcomes per 4-h increment in documented APP exposure. Subgroup and duration-response analyses were considered exploratory and were not used to infer causality.

Sensitivity analyses were performed to address pandemic-related temporal confounding, immortal-time and survivor bias, HFNC FiO₂ documentation, and competing risk in ICU discharge analysis. To address temporal confounding related to the COVID-19 pandemic and changes in ICU respiratory support practice over time, pandemic period was added to adjusted models as a three-level covariate: pre-pandemic, pandemic, and post-pandemic. Calendar year of ICU admission was also summarized descriptively to evaluate temporal distribution between groups. COVID-19 pneumonia judged to be the dominant cause of respiratory failure was excluded from the eligible cohort.

To address immortal-time and survivor bias related to the primary APP definition of cumulative APP duration ≥10 h during the first ICU week, the interval from ICU admission to first documented APP session was summarized, and early intubation or death before APP eligibility thresholds was reported. APP initiation-based sensitivity analyses classified patients according to first documented APP initiation, regardless of cumulative APP duration, rather than according to cumulative exposure ≥10 h. In addition, 24-h and 48-h landmark analyses were performed. For each landmark analysis, patients who were intubated or died before the landmark time point were excluded, exposure status was determined according to APP initiation by the landmark, and subsequent intubation and 28-day mortality were reassessed using adjusted logistic regression models with the same covariates as the primary model.

To address the reliability of PaO₂/FiO₂ ratio estimation in HFNC patients, the oxygenation trajectory analysis was repeated after restricting ABG observations to those with device-set FiO₂ documented within the prespecified ABG time window. ABG observations without verifiable device-set FiO₂ were excluded from this sensitivity analysis rather than being replaced by estimated FiO₂ values.

Missing data were handled using a complete-case approach for the primary analysis because eligibility required complete core EMR documentation, including ABG results, nursing flow sheets, respiratory therapy records, and chest imaging reports. Patients with missing core exposure, primary outcome, or propensity score covariate data were excluded before propensity score matching. For APP-specific documentation, sessions with verifiable start and end times were included in cumulative duration calculations, whereas sessions with missing start time, end time, or unclear duration were not counted toward cumulative APP duration and were classified as non-adherent in adherence analyses. No statistical imputation was performed because the final matched cohort had complete data for the prespecified primary outcomes and model covariates.

Primary statistical analyses were performed using SPSS 26.0 (IBM Corp.). Fine-Gray competing-risk analysis was performed using R software with the cmprsk package. Statistical significance was defined as a two-sided *α* < 0.05.

## Results

### Patient selection and baseline characteristics

From 168 screened ICU patients with pulmonary infiltrates and recent ICI exposure between January 2019 and September 2024, 132 met the clinical criteria for severe ICI-associated pneumonitis after exclusion of microbiologically confirmed infectious pneumonia and other dominant causes of respiratory failure. Propensity score matching (1:1) generated 60 matched pairs (*n* = 120). All baseline characteristics were balanced after matching (all post-matching SMD < 0.1; Table 1), with no significant differences in the matched cohort (all *p* > 0.05). Control group validation confirmed 98.5% adherence to non-prone positioning (IQR 96.2–99.3%).

**Table 1:** Baseline characteristics of patients with severe ICI-associated pneumonitis before and after propensity score matching.

Variables	Before matching			After matching			
	APP (*n* = 72)	Control (*n* = 60)	SMD	APP group (*n* = 60)	Control group (*n* = 60)	*p*-value	SMD
Demographics							
Age, years	62.4 ± 10.3	64.1 ± 9.8	0.17	63.2 ± 9.6	63.5 ± 10.1	0.87	0.03
Male sex, *n* (%)	44 (61.1)	35 (58.3)	0.06	37 (61.7)	36 (60.0)	0.85	0.03
BMI, kg/m^2^	26.3 ± 4.5	25.8 ± 5.1	0.10	26.0 ± 4.2	25.9 ± 4.8	0.91	0.02
Current smoker, *n* (%)	18 (25.0)	15 (25.0)	0.00	15 (25.0)	14 (23.3)	0.83	0.04
Alcohol use, *n* (%)	12 (16.7)	9 (15.0)	0.05	10 (16.7)	9 (15.0)	0.80	0.05
Malignancy profile							
Malignancy type, *n* (%)		0.12		0.92	0.02		
- Solid tumor	58 (80.6)	47 (78.3)		48 (80.0)	48 (80.0)		
- Hematologic	14 (19.4)	13 (21.7)		12 (20.0)	12 (20.0)		
Metastatic disease, *n* (%)	41 (56.9)	33 (55.0)	0.04	34 (56.7)	33 (55.0)	0.85	0.03
Comorbidities							
Hypertension, *n* (%)	35 (48.6)	28 (46.7)	0.04	29 (48.3)	28 (46.7)	0.86	0.03
Diabetes mellitus, *n* (%)	24 (33.3)	19 (31.7)	0.03	20 (33.3)	19 (31.7)	0.85	0.04
COPD, *n* (%)	13 (18.1)	10 (16.7)	0.04	11 (18.3)	10 (16.7)	0.81	0.04
Chronic kidney disease, *n* (%)	9 (12.5)	8 (13.3)	0.02	8 (13.3)	7 (11.7)	0.78	0.05
Immunotherapy parameters							
ICI class, *n* (%)		0.08		0.94	0.01		
- PD-1/PD-L1 inhibitors	51 (70.8)	42 (70.0)		43 (71.7)	42 (70.0)		
- CTLA-4 inhibitors	21 (29.2)	18 (30.0)		17 (28.3)	18 (30.0)		
Time from last ICI, days	28.5 ± 12.6	30.2 ± 14.1	0.13	29.1 ± 11.8	29.3 ± 13.4	0.92	0.02
Disease severity							
SOFA score	7.2 ± 2.1	7.5 ± 2.3	0.14	7.3 ± 2.0	7.3 ± 2.2	0.99	0.00
Baseline PaO₂/FiO₂, mmHg	158 ± 41	162 ± 38	0.10	160 ± 39	159 ± 40	0.89	0.03
Vasopressor use, *n* (%)	22 (30.6)	18 (30.0)	0.01	18 (30.0)	18 (30.0)	1.00	0.00
Pandemic period, *n* (%)			0.08			0.96	0.04
- Pre-pandemic	12 (16.7)	9 (15.0)		10 (16.7)	9 (15.0)		
- Pandemic	42 (58.3)	34 (56.7)		35 (58.3)	36 (60.0)		
- Post-pandemic	18 (25.0)	17 (28.3)		15 (25.0)	15 (25.0)		
Confounding interventions							
Respiratory support, *n* (%)			0.11			0.98	0.01
- HFNC	45 (62.5)	36 (60.0)		38 (63.3)	37 (61.7)		
- NIV	27 (37.5)	24 (40.0)		22 (36.7)	23 (38.3)		
Peak glucocorticoid dose*, mg/kg/day	1.8 ± 0.7	1.9 ± 0.8	0.13	1.8 ± 0.6	1.8 ± 0.7	0.91	0.02
Broad-spectrum antibiotics, *n* (%)	68 (94.4)	56 (93.3)	0.05	57 (95.0)	56 (93.3)	0.69	0.07

Data presented as mean ± standard deviation or *n* (%). *Peak methylprednisolone-equivalent dose within 72 h post-ICU admission. BMI: Body Mass Index; COPD: Chronic Obstructive Pulmonary Disease; HFNC: High-Flow Nasal Cannula; NIV: Non-Invasive Ventilation; ICI: Immune Checkpoint Inhibitor. Pre-matching comparisons by independent t-test (continuous) or χ^2^ test (categorical); post-matching by paired *t*-test (continuous) or McNemar’s test (categorical). Pandemic period was categorized as pre-pandemic (January 1, 2019 to December 31, 2019), pandemic (January 1, 2020 to December 31, 2022), and post-pandemic (January 1, 2023 to September 30, 2024). All post-matching SMD values <0.1 indicate excellent balance.

### PaO₂/FiO₂ ratio trajectories and physiological response

**Figure 1:**
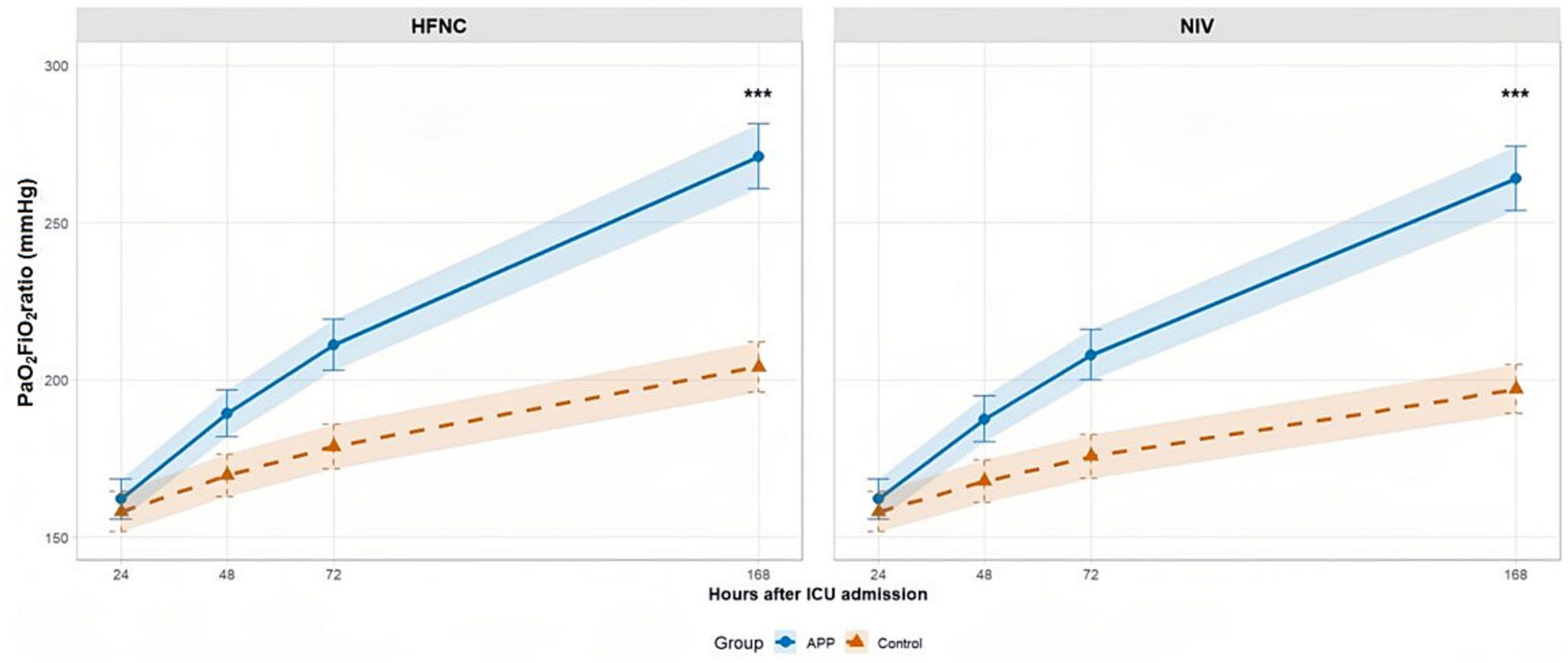
PaO₂/FiO₂ ratio trajectories over time in patients receiving different respiratory support modalities. Mean PaO₂/FiO₂ ratios with 95% confidence intervals are shown at 24, 48, 72, and 168 h after ICU admission for patients receiving high-flow nasal cannula (HFNC, left panel) and non-invasive ventilation (NIV, right panel). Blue solid lines represent the awake prone positioning (APP) group, and orange dashed lines represent the control group. Error bars indicate the standard error of the mean. ***p < 0.001 for the between-group comparison at 168 h.

The APP group demonstrated significantly greater improvement in PaO₂/FiO₂ ratio trajectories than controls over 168 h (Figure 1). At 24 h after ICU admission, the mean PaO₂/FiO₂ ratio was comparable between groups (APP: 162 ± 38 mmHg vs. control: 158 ± 41 mmHg, *p* = 0.47). Based on the mixed-effects model, by 168 h, the APP group had higher PaO₂/FiO₂ ratios in both respiratory support subgroups (HFNC: 271 mmHg vs. 204 mmHg; NIV: 264 mmHg vs. 197 mmHg; *p* < 0.001 for both comparisons). In the overall cohort, the PaO₂/FiO₂ ratio at 168 h reached 268 ± 52 mmHg in the APP group versus 201 ± 49 mmHg in controls (*p* < 0.001), representing a 65.4% mean increase from baseline in the overall APP group versus 27.2% in the overall control group (p < 0.001). The percentage improvements were consistent across respiratory support types, with HFNC patients showing 67.3% vs. 29.1% improvement and NIV patients showing 63.0% vs. 24.7% improvement in the APP and control groups, respectively, (*p* < 0.001 for all comparisons). Linear mixed-effects modeling revealed significant improvements in oxygenation dynamics favoring the APP group (global group × time interaction: *F*(3,354) = 27.3, *p* < 0.001). Key findings are summarized in Table 2, with complete model specifications below. Prone sessions yielded immediate physiological benefits, with a mean percentage increase in PaO₂/FiO₂ ratio of 23.4% ± 8.9% per session (95% CI: 20.1–26.7%, p < 0.001).

**Table 2:** Linear mixed-effects model for PaO₂/FiO₂ ratio dynamics.

Parameter	*β* Coefficient	95% CI	*p*-value
Main effects			
Group (APP vs. Control)	18.72	9.85–27.59	<0.001
Time (per 24 h increment)	15.39	12.07–18.71	<0.001
Interaction effects			
APP × 48 h	9.47	2.31–16.63	0.009
APP × 72 h	22.16	13.84–30.48	<0.001
APP × 168 h	44.29	34.15–54.43	<0.001
Covariates			
Respiratory support (NIV vs. HFNC)	−7.15	−12.83 to −1.47	0.014
Prone angle (per 1°)	0.89	0.31–1.47	0.003
Random Effects	Variance	95% CI	
Patient intercept	142.6	118.9–171.2	
Residual	38.9	34.1–44.3	

The linear mixed-effects model analyzed PaO₂/FiO₂ ratio trajectories with fixed effects for group, time (categorical: 24 h/48 h/72 h/168 h), group-time interaction, baseline respiratory support mode (HFNC/NIV), and mean prone positioning angle; random intercepts accounted for within-patient correlations using unstructured covariance (optimal per BIC). Reference categories: Control group for group effect, HFNC for respiratory support, 24 h for time. Model diagnostics confirmed residuals normality (Shapiro–Wilk p = 0.18), homoscedasticity (Breusch-Pagan *p* = 0.19), and absence of multicollinearity (max VIF = 1.8). Fit indices: AIC = 3,247, BIC = 3,312; marginal R^2^ = 0.42 (fixed effects), conditional R^2^ = 0.78 (total variance explained). Coefficients represent mean change in PaO₂/FiO₂ ratio, expressed in mmHg, with 95% CI.

### Factors associated with endotracheal intubation risk

Univariate analysis identified significant associations between intubation and group allocation (APP 18.3% vs. control 45.0%; χ^2^ = 10.24, *p* = 0.001), respiratory support mode, and clinical severity markers (Table 3). The final multivariable model included all prespecified covariates plus clinically relevant predictors identified in univariate screening, including baseline PaO₂/FiO₂ ratio ≤200 mmHg and vasopressor use. After adjustment, APP was associated with lower intubation risk (adjusted OR = 0.24, 95% CI, 0.10–0.59; *p* = 0.002), while NIV versus HFNC, vasopressor dependence, and severe baseline hypoxemia, defined as PaO₂/FiO₂ ratio ≤200 mmHg, were associated with higher intubation risk (Table 4). Model intercept (*β* = −3.21, SE = 0.89) reflected baseline risk consistent with cohort severity.

**Table 3:** Univariate analysis of intubation risk factors.

Variable	Intubated	Non-intubated	χ^2^/Fisher	*p*-value	Risk difference (95% CI)
Group allocation			10.24	0.001	−26.7% (−42.1 to −11.2%)
APP group	11/60 (18.3%)	49/60 (81.7%)			
Control group	27/60 (45.0%)	33/60 (55.0%)			
Respiratory support			6.87	0.009	27.6% (9.8 to 45.4%)
NIV	22/45 (48.9%)	23/45 (51.1%)			
HFNC	16/75 (21.3%)	59/75 (78.7%)			
Baseline PaO₂/FiO₂ ratio ≤200 mmHg	31/79 (39.2%)	48/79 (60.8%)	6.15	0.013	24.6% (5.3 to 43.9%)
Baseline PaO₂/FiO₂ ratio >200 mmHg	7/41 (17.1%)	34/41 (82.9%)			
Vasopressor use			10.29	0.001	29.3% (10.6 to 48.0%)
Yes	19/36 (52.8%)	17/36 (47.2%)			
No	19/84 (22.6%)	65/84 (77.4%)			

Data are presented as n/total (%), using row percentages within each exposure category. Risk differences were calculated as the intubation rate in the exposed or higher-risk category minus the reference category, except for group allocation, where the risk difference was calculated as APP group minus control group. APP, awake prone positioning; HFNC, high-flow nasal cannula; NIV, non-invasive ventilation; PaO₂/FiO₂, arterial oxygen partial pressure to fraction of inspired oxygen ratio.

**Table 4:** Multivariable logistic regression for intubation risk.

Predictor	*β*	SE	Wald χ^2^	df	aOR (95% CI)	*p*-value
Primary exposure						
APP group (vs. control)	−1.427	0.452	9.97	1	0.24 (0.10–0.59)	0.002
Core adjustments						
Age (per 5-year increase)	0.192	0.108	3.18	1	1.21 (0.98–1.50)	0.075
SOFA score (per point)	0.361	0.098	13.58	1	1.43 (1.18–1.74)	<0.001
ICI class (CTLA-4 vs. PD-1)	0.683	0.482	2.01	1	1.98 (0.77–5.09)	0.156
Peak glucocorticoid* (mg/kg/day)	−0.289	0.201	2.07	1	0.75 (0.50–1.11)	0.150
Clinically relevant covariates						
Baseline PaO₂/FiO₂ ratio ≤200 mmHg	1.103	0.476	5.37	1	3.01 (1.19–7.67)	0.020
Respiratory support (NIV vs. HFNC)	1.025	0.438	5.48	1	2.79 (1.18–6.58)	0.019
Vasopressor dependence	1.217	0.456	7.13	1	3.38 (1.38–8.27)	0.008

*Peak methylprednisolone-equivalent dose within 72 h post-ICU admission. Reference categories: Control group for APP; HFNC for respiratory support; PD-1 for ICI class; Baseline PaO₂/FiO₂ ratio >200 mmHg for baseline oxygenation. Model Summary: −2 Log Likelihood = 146.32; Hosmer-Lemeshow χ^2^ = 4.73 (p = 0.69); Nagelkerke R^2^ = 0.52. Abbreviations: aOR, adjusted odds ratio; SE, standard error; df, degrees of freedom.

### Time-to-discharge analysis of ICU length of sta

**Figure 2:**
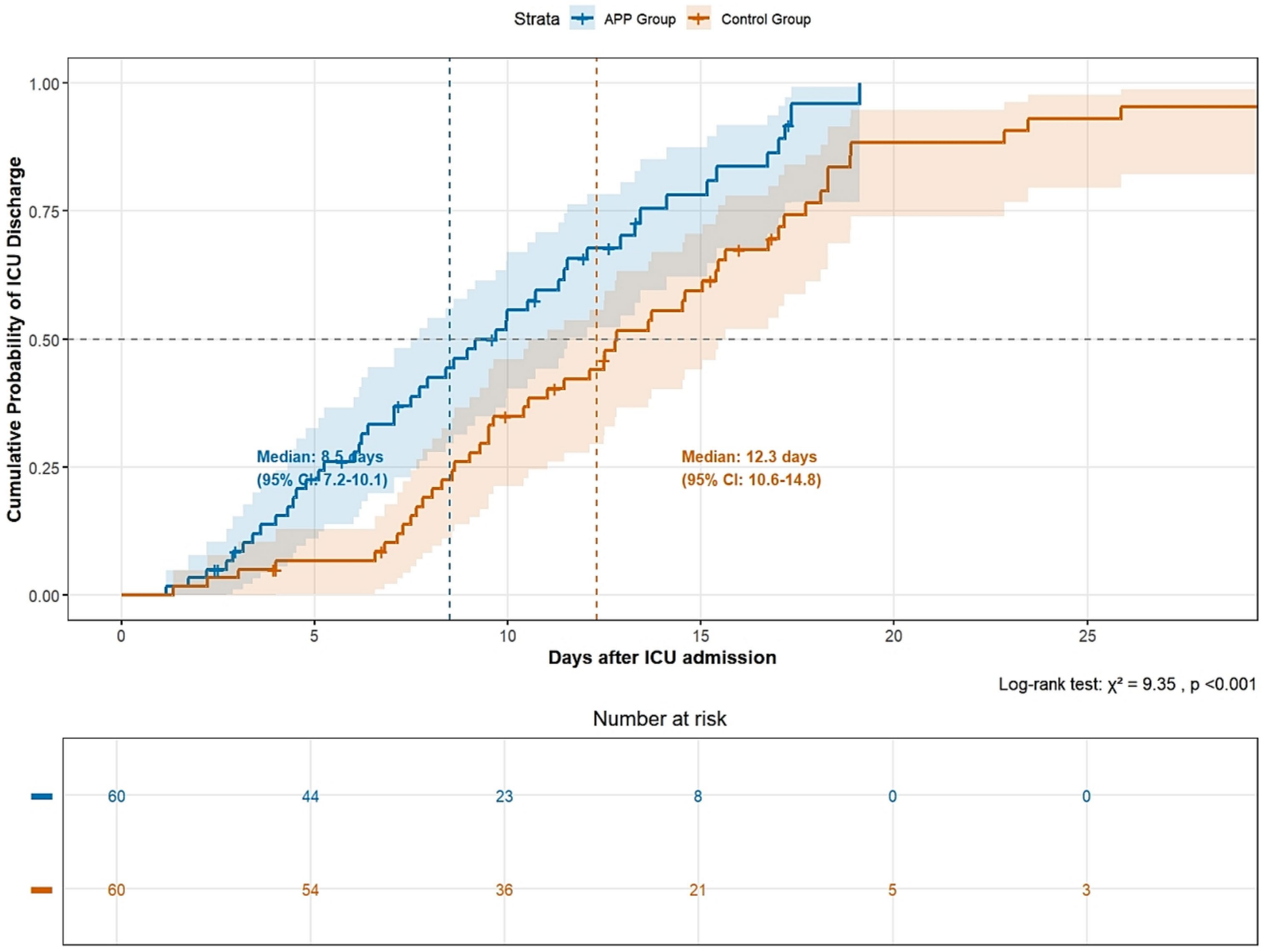
Time-to-discharge analysis: Kaplan–Meier curves for ICU discharge. Kaplan–Meier curves show the cumulative probability of ICU discharge over time for patients in the APP group (blue line) and control group (orange line). Shaded areas represent 95% confidence intervals. Vertical dashed lines indicate the median time to ICU discharge for each group. The horizontal dashed line at 0.5 probability intersects with the vertical lines at the median discharge times. The “Number at risk” table displays the number of patients remaining in the ICU at 0, 5, 10, 15, 20, and 25 days after admission. The log-rank test demonstrated a significant difference between groups (χ^2^ = 9.35; *p* < 0.001).

Kaplan–Meier analysis demonstrated significantly shorter ICU stays in the APP group (median 8.5 days, 95% CI: 7.2–10.1) versus controls (median 12.3 days, 95% CI: 10.6–14.8; log-rank χ^2^ = 9.35, *p* < 0.001) (Figure 2). Stratified Cox proportional hazards modeling (adjusted for age, SOFA score, ICI class, peak glucocorticoid dose, vasopressor dependence, and respiratory support mode) confirmed APP as an independent predictor of accelerated ICU discharge (adjusted HR = 2.08, 95% CI: 1.40–3.09, p < 0.001), with significant associations also observed for SOFA score (HR = 0.86, 95% CI: 0.80–0.93, p < 0.001), vasopressor dependence (HR = 0.58, 95% CI: 0.39–0.87, *p* = 0.009), and respiratory support mode (NIV vs. HFNC: HR = 0.67, 95% CI: 0.46–0.97, *p* = 0.033), while proportional hazards assumptions were validated by non-significant global Schoenfeld test (χ^2^ = 6.54, *p* = 0.48) (Table 5).

**Table 5:** Stratified cox proportional hazards model for ICU discharge.

Predictor	*β*	SE	Wald χ^2^	df	Adjusted HR (95% CI)	*p*-value
Primary exposure						
APP group (vs. control)	0.732	0.201	13.28	1	2.08 (1.40–3.09)	<0.001
Stratification factor						
Baseline PaO₂/FiO₂ ratio ≤200 mmHg	-	-	-	-	Stratum variable	-
Adjustment covariates						
Age (per 5-year increase)	−0.102	0.051	4.01	1	0.90 (0.82–1.00)	0.045
SOFA score (per point)	−0.150	0.039	14.79	1	0.86 (0.80–0.93)	<0.001
ICI class (CTLA-4 vs. PD-1)	−0.210	0.193	1.18	1	0.81 (0.55–1.19)	0.278
Peak glucocorticoid* (mg/kg/day)	−0.118	0.103	1.31	1	0.89 (0.73–1.09)	0.252
Clinically relevant covariates						
Vasopressor dependence	−0.538	0.205	6.89	1	0.58 (0.39–0.87)	0.009
Respiratory support (NIV vs. HFNC)	−0.402	0.188	4.57	1	0.67 (0.46–0.97)	0.033

*Peak methylprednisolone-equivalent dose within 72 h post-ICU admission. Event definition: ICU discharge order. Censoring: death or transfer to other ICU. Proportional hazards assumption validated by: non-significant global Schoenfeld test (*p* = 0.48); Covariate-specific tests all *p* > 0.10; Log-minus-log plots parallelism. Model diagnostics: global Schoenfeld test: χ^2^ = 6.54, *p* = 0.48; −2 Log likelihood = 352.71.

### Factors associated with 28-day all-cause mortality

In the matched cohort, 32 of 120 patients died within 28 days after ICU admission. The 28-day all-cause mortality rate was 18.3% (11/60) in the APP group and 35.0% (21/60) in the control group (χ^2^ = 4.26, *p* = 0.039), corresponding to an absolute risk difference of −16.7% (95% CI, −32.2 to −1.1%) (Table 6). Univariate analysis also showed that 28-day mortality was associated with vasopressor use, respiratory support mode, and SOFA score >7 (Table 6). In multivariable logistic regression adjusted for age, SOFA score, ICI class, peak glucocorticoid dose, vasopressor dependence, and respiratory support mode, APP remained associated with lower odds of 28-day all-cause mortality (adjusted OR = 0.32, 95% CI, 0.13–0.81; *p* = 0.015) (Table 7). Significant mortality-associated factors included baseline SOFA score (adjusted OR = 1.48 per point, 95% CI, 1.18–1.85; *p* < 0.001) and vasopressor dependence (adjusted OR = 3.15, 95% CI, 1.24–8.02; *p* = 0.017) (Table 7).

**Table 6:** Univariate analysis of 28-day mortality risk factors.

Variable	Died	Survived	χ^2^/Fisher	*p*-value	Risk difference (95% CI)
Group allocation			4.26	0.039	−16.7% (−32.2 to −1.1%)
APP group	11/60 (18.3%)	49/60 (81.7%)			
Control group	21/60 (35.0%)	39/60 (65.0%)			
Vasopressor use			9.29	0.002	26.4% (8.6 to 44.3%)
Yes	17/38 (44.7%)	21/38 (55.3%)			
No	15/82 (18.3%)	67/82 (81.7%)			
Respiratory support			8.91	0.003	24.9% (8.1 to 41.7%)
NIV	19/45 (42.2%)	26/45 (57.8%)			
HFNC	13/75 (17.3%)	62/75 (82.7%)			
SOFA score >7			8.35	0.004	23.3% (8.1 to 38.6%)
Yes	23/60 (38.3%)	37/60 (61.7%)			
No	9/60 (15.0%)	51/60 (85.0%)			
Age >65 years			0.68	0.409	6.7% (−9.1 to 22.4%)
Yes	18/60 (30.0%)	42/60 (70.0%)			
No	14/60 (23.3%)	46/60 (76.7%)			
ICI class			0.61	0.436	6.6% (−10.3 to 23.5%)
CTLA-4 inhibitors	13/42 (31.0%)	29/42 (69.0%)			
PD-1/PD-L1 inhibitors	19/78 (24.4%)	59/78 (75.6%)			

Data are presented as n/total (%), using row percentages within each exposure category. Risk differences were calculated as the mortality rate in the exposed or higher-risk category minus the reference category, except for group allocation, where the risk difference was calculated as APP group minus control group. Pearson χ^2^ tests were used for categorical comparisons. APP, awake prone positioning; CTLA-4, cytotoxic T-lymphocyte-associated protein 4; HFNC, high-flow nasal cannula; ICI, immune checkpoint inhibitor; NIV, non-invasive ventilation; PD-1, programmed death-1; PD-L1, programmed death ligand-1; SOFA, Sequential Organ Failure Assessment.

**Table 7:** Multivariable logistic regression for 28-day all-cause mortality.

Predictor	*β*	SE	Wald χ^2^	df	Adjusted OR (95% CI)	*p*-value
Primary exposure						
APP group (vs. control)	−1.139	0.470	5.87	1	0.32 (0.13–0.81)	0.015
Core adjustments						
Age (per 5-year increase)	0.201	0.120	2.80	1	1.22 (0.97–1.55)	0.094
SOFA score (per point)	0.392	0.103	14.52	1	1.48 (1.18–1.85)	<0.001
ICI class (CTLA-4 vs. PD-1)	0.305	0.510	0.36	1	1.36 (0.50–3.68)	0.550
Peak glucocorticoid* (mg/kg/day)	−0.185	0.222	0.69	1	0.83 (0.54–1.28)	0.406
Clinically relevant covariates						
Vasopressor dependence	1.147	0.482	5.66	1	3.15 (1.24–8.02)	0.017
Respiratory support (NIV vs. HFNC)	0.621	0.458	1.84	1	1.86 (0.76–4.56)	0.175

*Peak methylprednisolone-equivalent dose within 72 h post-ICU admission. Reference categories: Control group for APP; HFNC for respiratory support; PD-1 inhibitors for ICI class. Model Summary: Hosmer-Lemeshow test: χ^2^ = 6.28, *p* = 0.62 (goodness-of-fit); Nagelkerke R^2^ = 0.41; Overall prediction accuracy: 84.2%.

### Sensitivity analyses addressing temporal confounding, APP exposure timing, FiO₂ documentation, and competing risk

Several sensitivity analyses were conducted to evaluate whether the primary findings were affected by pandemic-related temporal confounding, APP exposure timing, FiO₂ documentation, or competing risk in ICU discharge analysis. COVID-19 pneumonia judged as the dominant cause of acute hypoxemic respiratory failure was excluded before cohort assembly. SARS-CoV-2 testing or clinician-documented COVID-19 diagnostic assessment was available in 151 of 168 screened patients (89.9%); testing was not uniformly available in early 2019 but was reviewed when clinically performed and after the emergence of COVID-19. Seven patients were excluded before final cohort assembly because SARS-CoV-2 infection or COVID-19 pneumonia was considered the dominant cause of respiratory failure. In the matched cohort, the distribution of pandemic period was balanced between the APP and control groups (pre-pandemic: 10/60 [16.7%] vs. 9/60 [15.0%]; pandemic: 35/60 [58.3%] vs. 36/60 [60.0%]; post-pandemic: 15/60 [25.0%] vs. 15/60 [25.0%]; *p* = 0.96). In the pre-matching cohort, the proportion of patients receiving APP did not differ significantly across the pre-pandemic, pandemic, and post-pandemic periods (57.1, 55.3, and 51.4%, respectively; *p* = 0.89). After additional adjustment for pandemic period, the associations between APP and intubation, 28-day mortality, and ICU discharge remained directionally consistent with the primary analyses (Supplementary Table S1).

To evaluate potential immortal-time and survivor bias related to the primary APP exposure definition, the timing of APP initiation was examined. Among the 60 patients in the APP group, the median time from ICU admission to the first documented APP session was 13 h (IQR, 8–21 h). APP was initiated within 24 h in 46 patients (76.7%) and within 48 h in 56 patients (93.3%). Before the 24-h landmark, 6 patients were intubated and 1 patient died; these 7 patients were excluded from the 24-h landmark analysis. Before the 48-h landmark, 12 patients were intubated and 3 patients died; these 15 patients were excluded from the 48-h landmark analysis. In the eligible pre-matching source cohort, 5 patients had documented APP initiation but were intubated or died before accumulating 10 h of APP exposure and therefore did not meet the primary cumulative APP exposure definition. In the APP initiation-based sensitivity analysis, which used first documented APP initiation rather than cumulative APP duration ≥10 h as the exposure definition, APP initiation remained associated with lower odds of intubation and showed a directionally consistent association with lower 28-day mortality (Supplementary Table S1). The 24-h and 48-h landmark analyses showed attenuated but directionally consistent estimates for both intubation and 28-day mortality, indicating that the primary findings were not explained solely by survival long enough to accumulate ≥10 h of APP exposure.

For the PaO₂/FiO₂ ratio trajectory analysis, device-set FiO₂ was available for 438 of 480 scheduled ABG-FiO₂ pairs (91.3%) in the matched cohort. Among HFNC observations, device-set FiO₂ was available for 275 of 300 ABG-FiO₂ pairs (91.7%). In the sensitivity analysis restricted to ABG measurements with documented device-set FiO₂, the group × time interaction remained significant (*F* = 24.8, *p* < 0.001), and the APP group continued to show a higher PaO₂/FiO₂ ratio at 168 h than the control group (p < 0.001). These findings were consistent with the primary oxygenation trajectory analysis (Supplementary Table S1).

In the primary time-to-discharge analysis, death before ICU discharge was censored. Because death represents an informative competing event, a Fine-Gray competing-risk sensitivity analysis was performed with death before ICU discharge treated as the competing event. In this model, APP remained associated with a higher cumulative incidence of ICU discharge (subdistribution HR = 1.82, 95% CI, 1.23–2.69; *p* = 0.003), consistent with the direction of the primary Cox model, although the effect estimate was attenuated (Supplementary Table S1).

### Exploratory subgroup and duration-response analyses

Exploratory stratified analysis by baseline oxygenation status suggested that the association between APP and intubation risk differed according to baseline PaO₂/FiO₂ ratio. Among patients with severe baseline hypoxemia, defined as PaO₂/FiO₂ ratio ≤200 mmHg (*n* = 79), APP was associated with lower intubation risk (adjusted OR = 0.18, 95% CI, 0.06–0.53; *p* = 0.002), whereas the association was not statistically significant among patients with PaO₂/FiO₂ ratio >200 mmHg (*n* = 41; adjusted OR = 0.42, 95% CI, 0.12–1.48; *p* = 0.178; interaction *p* = 0.039). Similarly, the association between APP and lower 28-day mortality was observed mainly in the severe hypoxemia subgroup (adjusted OR = 0.21, 95% CI, 0.07–0.62; *p* = 0.005), whereas this association was not statistically significant in patients with PaO₂/FiO₂ ratio >200 mmHg (adjusted OR = 0.68, 95% CI, 0.15–3.01; *p* = 0.613; interaction *p* = 0.042). When stratified by baseline respiratory support modality, APP was associated with lower intubation risk in both the HFNC subgroup (adjusted OR = 0.27, 95% CI, 0.09–0.80; *p* = 0.018) and the NIV subgroup (adjusted OR = 0.19, 95% CI, 0.04–0.82; *p* = 0.026), with no statistically significant interaction by respiratory support mode (interaction *p* = 0.623). The associations with 28-day mortality were directionally consistent but did not reach statistical significance within either respiratory support subgroup (HFNC: adjusted OR = 0.35, 95% CI, 0.11–1.08; *p* = 0.067; NIV: adjusted OR = 0.29, 95% CI, 0.08–1.11; *p* = 0.071; interaction *p* = 0.791). In exploratory duration-response analysis, each additional 4-h increment in documented cumulative APP duration was associated with lower odds of intubation (adjusted OR = 0.83, 95% CI, 0.72–0.95; *p* = 0.009) and 28-day mortality (adjusted OR = 0.87, 95% CI, 0.77–0.99; *p* = 0.037) after adjustment for the same covariates. These findings should be interpreted as exploratory associations rather than evidence of a causal dose–response effect. Table 8 summarizes these exploratory subgroup and duration-response analyses.

**Table 8:** Exploratory subgroup and duration-response analyses of APP-associated clinical outcomes.

Subgroup	*n*	Intubation outcome		28-day mortality outcome	
		Adjusted OR (95% CI)	*p*-value	Adjusted OR (95% CI)	*p*-value
By baseline oxygenation					
PaO₂/FiO₂ ratio ≤200 mmHg	79	0.18 (0.06–0.53)	0.002	0.21 (0.07–0.62)	0.005
PaO₂/FiO₂ ratio >200 mmHg	41	0.42 (0.12–1.48)	0.178	0.68 (0.15–3.01)	0.613
Interaction *p*-value		0.039		0.042	
By respiratory support					
HFNC	75	0.27 (0.09–0.80)	0.018	0.35 (0.11–1.08)	0.067
NIV	45	0.19 (0.04–0.82)	0.026	0.29 (0.08–1.11)	0.071
Interaction *p*-value		0.623		0.791	
By cumulative prone duration					
Per 4-h increment	-	0.83 (0.72–0.95)	0.009	0.87 (0.77–0.99)	0.037
By APP protocol adherence					
High adherence (≥30° angle)	42	0.15 (0.05–0.49)	0.002	0.24 (0.08–0.71)	0.010
Moderate adherence (<30° angle)	18	0.39 (0.11–1.38)	0.144	0.52 (0.14–1.89)	0.319
Interaction *p*-value		0.046		0.173	

Subgroup and duration-response analyses were exploratory. Models were adjusted for age, SOFA score, ICI class, peak glucocorticoid dose, vasopressor dependence, and respiratory support mode, except for analyses stratified by respiratory support mode. Interaction terms were tested in separate models with identical covariates. For cumulative APP duration, odds ratios represent associations per 4-h increment in documented APP exposure and should not be interpreted as causal dose–response effects. High adherence was defined as documented positioning angle or head-of-bed elevation ≥30° with ≥2 h per session and cumulative APP duration ≥10 h during the first ICU week. APP, awake prone positioning; HFNC, high-flow nasal cannula; ICI, immune checkpoint inhibitor; NIV, non-invasive ventilation; PaO₂/FiO₂, arterial oxygen partial pressure to fraction of inspired oxygen ratio; SOFA, Sequential Organ Failure Assessment.

### Safety outcomes

**Figure 3:**
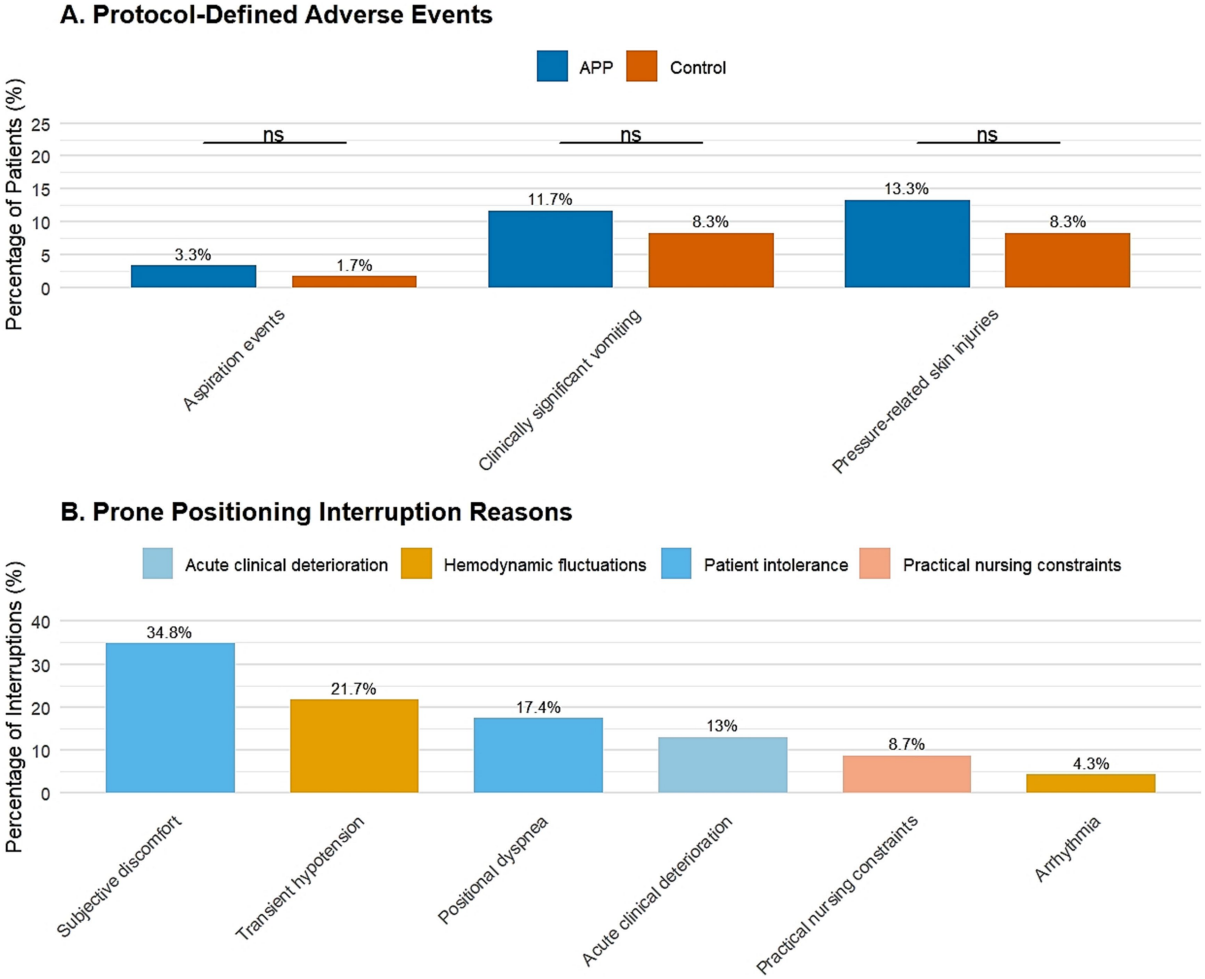
Safety outcomes and reasons for awake prone positioning interruptions. **(A)** Protocol-defined adverse events in the APP group (blue) and control group (orange). Data are expressed as the percentage of patients experiencing each event. Bars represent group proportions, and annotations indicate between-group statistical significance (ns, not significant). **(B)** Distribution of reasons for APP interruptions, categorized as patient intolerance, hemodynamic fluctuations, acute clinical deterioration, or practical nursing constraints. Values represent percentages of total recorded APP interruptions.

Protocol-defined adverse events were systematically monitored in both groups, and the overall safety profile was comparable between the APP and control groups (Figure 3). Pressure-related skin injuries occurred in 8 patients (13.3%) in the APP group versus 5 patients (8.3%) in the control group (*p* = 0.378), all classified as Stage 2 pressure injuries per National Pressure Injury Advisory Panel criteria. The most frequent anatomical locations in the APP group were the anterior chest (*n* = 4), chin (*n* = 3), and iliac crest (*n* = 2), while control group injuries predominantly affected the sacrum (*n* = 3) and heels (*n* = 2). All skin injuries resolved with standard wound care without progression to higher stages. Clinically significant vomiting was documented in 7 patients (11.7%) in the APP group compared to 5 patients (8.3%) in the control group (*p* = 0.543), with no significant difference in antiemetic requirements between groups (*p* = 0.612). Aspiration events were rare and comparable between groups (*p* = 0.559), with all cases classified as minor based on radiographic and clinical criteria.

Among the 60 patients in the APP group, 23 (38.3%) experienced at least one episode of prone positioning interruption before the planned session completion. The most common reasons for interruption were patient intolerance (52.2% of interruptions), primarily due to subjective discomfort (*n* = 8) and positional dyspnea (*n* = 4), followed by hemodynamic fluctuations (26.1%), predominantly characterized by transient hypotension (SBP < 90 mmHg) that resolved upon position change. Other interruption causes included acute clinical deterioration requiring urgent medical intervention (13.0%) and practical nursing constraints such as required procedures or imaging studies (8.7%). The mean interruption rate was 0.42 ± 0.18 per patient-day, with significant decline over sequential ICU days (*p* < 0.001), suggesting improved tolerance with repeated exposure. Notably, 78.3% of interrupted sessions were successfully resumed after temporary return to supine position, with median interruption duration of 47 min (IQR: 28–95).

## Discussion

This propensity-matched retrospective cohort study found that APP was associated with improved PaO₂/FiO₂ ratio trajectories, lower intubation rates, shorter ICU stay, and lower observed 28-day mortality among patients with clinically diagnosed severe ICI-associated pneumonitis and acute hypoxemic respiratory failure. These findings suggest that APP may be a feasible adjunctive respiratory support strategy in this specific oncologic population after microbiologically confirmed infectious pneumonia, COVID-19 pneumonia, and other dominant alternative causes of respiratory failure have been excluded. However, available APP evidence is derived predominantly from COVID-19-related acute hypoxemic respiratory failure or broader acute respiratory failure cohorts rather than from patients receiving ICI therapy. Therefore, comparisons with previous studies should be interpreted as indirect and hypothesis-generating (5, 10).

APP has been most extensively studied in COVID-19-related acute hypoxemic respiratory failure. Systematic reviews and randomized evidence have generally suggested that APP is associated with improved oxygenation and, in selected settings, lower intubation risk, although the magnitude of association has varied across studies and mortality effects have been less consistent (5, 11, 12). The multinational meta-trial by Ehrmann et al. (11) supported APP in non-intubated patients with COVID-19 acute hypoxemic respiratory failure receiving HFNC, whereas the randomized trial by Alhazzani et al. (13) showed a directionally favorable but statistically non-significant difference in intubation. Rosén et al. (14) also reported neutral findings in a multicenter randomized trial, highlighting that APP outcomes may depend on patient selection, respiratory support mode, adherence, and implementation fidelity. These heterogeneous findings are relevant to the present study because they support biological plausibility but do not directly establish the effect of APP in severe ICI-associated pneumonitis.

Compared with COVID-19 cohorts, patients with severe ICI-associated pneumonitis represent a distinct population. Respiratory failure in this setting is driven primarily by immune-mediated alveolar and interstitial inflammation, often in patients with active malignancy, recent ICI exposure, corticosteroid therapy, diagnostic uncertainty, and complex oncologic treatment decisions. These features differ from viral pneumonia cohorts in terms of disease biology, immunosuppression, competing diagnoses, and thresholds for escalation of respiratory support. Therefore, prior APP evidence from COVID-19 or general acute hypoxemic respiratory failure should be considered supportive but indirect when applied to ICI-treated cancer patients.

The oxygenation findings of the present study are consistent with the physiological rationale for prone positioning. APP may improve oxygenation through dependent lung recruitment, better ventilation-perfusion matching, and redistribution of regional ventilation. In the present cohort, the association between APP and improved PaO₂/FiO₂ trajectories is physiologically plausible, but should still be interpreted in light of retrospective exposure classification and the possibility that patients who tolerated APP were clinically more stable.

The lower intubation rate observed in the APP group is broadly compatible with prior APP literature, but the external comparison remains indirect. Meta-analytic evidence in COVID-19 suggests that APP is more likely to be associated with lower intubation risk in patients receiving advanced respiratory support or treated in ICU settings (5, 12). Secondary analyses have suggested that APP success may be related to patient tolerance, oxygenation response, respiratory rate response, and ability to sustain prone-positioning exposure (15). International prospective data have also suggested that APP-induced changes in oxygenation and respiratory rate are associated with subsequent intubation risk in COVID-19-related acute hypoxemic respiratory failure (16). These findings are relevant because patients with severe ICI-associated pneumonitis frequently require HFNC or NIV, but the present cohort differs from COVID-19 cohorts in immunologic lung injury, cancer burden, corticosteroid treatment, and clinical escalation thresholds. Thus, the observed association with lower intubation should be presented as consistent with, rather than directly proven by, prior APP evidence.

APP exposure and adherence deserve specific attention. Prior trials and meta-analyses indicate that actual prone duration varies widely, and that longer or better-tolerated APP exposure may be associated with more favorable outcomes in selected patients (12, 15). However, duration-based analyses are also vulnerable to survivor bias and tolerance bias, because patients who remain stable and cooperative have more opportunity to accumulate longer APP exposure. In the present study, sensitivity analyses using APP initiation rather than cumulative APP duration, together with 24-h and 48-h landmark analyses, showed attenuated but directionally consistent estimates. These analyses reduce, but do not eliminate, concern that the primary APP definition may have overestimated the association between APP and clinical outcomes.

In terms of ICU length of stay, the shorter ICU stay observed in the APP group may reflect a combination of improved oxygenation trajectory, lower observed intubation rate, and earlier clinical stabilization. However, previous APP studies have not consistently demonstrated shorter ICU or hospital length of stay (12, 13). In the present study, the competing-risk sensitivity analysis supported the direction of the primary ICU discharge analysis, but death before ICU discharge remains clinically informative and should be considered when interpreting time-to-discharge outcomes. From a resource perspective, shorter ICU stay may have implications for ICU bed turnover and allocation in settings with constrained capacity, but this study did not perform a formal cost-effectiveness analysis and did not collect detailed cost, staffing, or hospital-level bed utilization data.

The observed association between APP and lower 28-day mortality should be interpreted more cautiously than the oxygenation and intubation findings. Randomized and meta-analytic APP evidence has been more consistent for intubation or treatment failure than for mortality (11–13). In retrospective cohorts, mortality associations may be influenced by unmeasured illness severity, clinician selection, performance status, cooperation, hemodynamic stability, and tolerance of the intervention. In the present study, PSM, multivariable adjustment, pandemic-period adjustment, landmark analyses, and APP-initiation sensitivity analyses were used to reduce bias, but residual confounding remains likely. Therefore, the mortality finding should be viewed as hypothesis-generating rather than evidence of definitive efficacy.

Safety and tolerance are central to APP implementation. In randomized and meta-analytic APP evidence, serious adverse events have generally been uncommon, but discomfort, musculoskeletal symptoms, desaturation, device displacement, pressure-related skin complications, and intolerance may limit adherence (12–14). Pressure-related skin injury is a known complication of prone positioning and requires active prevention, regular skin assessment, pressure-relief materials, and nursing surveillance (8). In the present study, protocol-defined adverse events were broadly comparable between groups, while APP interruptions were common and were mainly related to intolerance, positional dyspnea, and transient hemodynamic fluctuation. Olmos et al. (17) reported that a structured bundle of care was associated with improved tolerance of APP in patients with acute respiratory failure, supporting the relevance of nursing interventions, comfort measures, and repeated tolerance assessment. These observations indicate that APP feasibility depends not only on respiratory physiology but also on bedside monitoring, patient cooperation, pressure-injury prevention, and nursing capacity.

Several limitations should be considered. First, this was a single-center retrospective study, and selection bias and residual confounding cannot be fully excluded despite PSM, multivariable adjustment, and sensitivity analyses. Although measured baseline variables, including SOFA score, baseline PaO₂/FiO₂ ratio, respiratory support mode, vasopressor dependence, malignancy type, ICI class, glucocorticoid exposure, and pandemic period, were balanced or adjusted for, APP feasibility also depends on factors incompletely captured in retrospective records. Patients who received APP may have been more awake, cooperative, hemodynamically stable, and able to tolerate prolonged positioning, whereas patients who did not receive APP may have had delirium, agitation, impaired consciousness, poor performance status, aspiration risk, positional intolerance, immediate need for intubation, or limited ability to cooperate. The exploratory duration-response analysis of cumulative APP exposure is also susceptible to survivor bias and tolerance bias. Accordingly, the present findings should be interpreted as hypothesis-generating rather than definitive evidence of causality.

Second, ICI-associated pneumonitis is a clinical diagnosis of exclusion and lacks a single definitive biomarker. Although we used structured physician adjudication, imaging review, and exclusion of microbiologically confirmed infectious pneumonia, COVID-19 pneumonia, opportunistic infection, tumor progression, pulmonary edema, pulmonary embolism, radiation pneumonitis, and diffuse alveolar hemorrhage when these were considered dominant causes, residual diagnostic misclassification remains possible. Third, the external APP evidence used for comparison is indirect because it is derived predominantly from COVID-19-related acute hypoxemic respiratory failure or broader acute respiratory failure populations rather than from ICI-treated cancer patients. Fourth, APP exposure was delivered in real-world clinical practice within an institutional ICU positioning framework rather than as a prospectively standardized fixed intervention protocol. Daily APP duration, session frequency, positioning angle maintenance, interruptions, and patient tolerance varied according to clinical status and bedside feasibility. Finally, long-term outcomes, including post-ICU functional recovery, pulmonary function, quality of life, oncologic treatment continuation after recovery, and ICU readmission after discharge, were not captured. No formal cost-effectiveness analysis was performed, and data on ICU costs, nursing workload, staffing requirements, and hospital-level bed utilization were not systematically collected.

Future prospective multicenter studies should use standardized diagnostic adjudication for ICI-associated pneumonitis, predefined APP initiation and discontinuation criteria, prespecified APP exposure targets, and systematic recording of feasibility-related factors. Such studies should collect data on consciousness level, delirium or agitation, cooperation, performance status, patient tolerance, clinician decision-making, nursing workload, staffing availability, APP duration, positioning angle, interruptions, and reasons for non-initiation or discontinuation. Randomized or pragmatic trial designs would be particularly valuable to reduce confounding by indication and clarify whether APP has an independent effect on intubation, ICU length of stay, and mortality in this population.

In conclusion, this study provides preliminary evidence that awake prone positioning may be a feasible adjunctive respiratory support strategy for patients with clinically diagnosed severe ICI-associated pneumonitis and acute hypoxemic respiratory failure. In this retrospective propensity-matched cohort, APP was associated with improved oxygenation, lower intubation rates, shorter ICU stay, and lower observed 28-day mortality. However, the mortality and subgroup findings should be interpreted cautiously because of the observational design, single-center setting, limited sample size, indirectness of external evidence, and potential residual confounding. These findings are hypothesis-generating and require confirmation in prospective multicenter studies focused specifically on ICI-associated pneumonitis.

## Data Availability

The original contributions presented in this study are included in the article and the Supplementary material. Further inquiries can be directed to the corresponding authors.
