# Supplementary material for: Association of awake prone positioning with clinical outcomes in severe immune checkpoint inhibitor-associated pneumonitis with acute hypoxemic respiratory failure: a propensity-matched retrospective cohort study
Source: Front Med (Lausanne). 2026 Jul 31;13:1880206. doi: 10.3389/fmed.2026.1880206 (PMC13473375; doi:10.3389/fmed.2026.1880206)
Supplement: Supplementary file 1 [file Table_1.DOCX]

**Supplementary Table S1.** Sensitivity analyses addressing temporal confounding, APP exposure timing, FiO₂ documentation, and competing risk

| **Analysis** | **Outcome** | **Model or restriction** | **Effect estimate for APP** | **p-value** | **Interpretation** |
| --- | --- | --- | --- | --- | --- |
| Calendar-period adjusted model | Endotracheal intubation | Logistic regression additionally adjusted for pandemic period | Adjusted OR = 0.25 (95% CI, 0.10–0.61) | 0.003 | Consistent with primary analysis |
| Calendar-period adjusted model | 28-day all-cause mortality | Logistic regression additionally adjusted for pandemic period | Adjusted OR = 0.34 (95% CI, 0.14–0.84) | 0.020 | Consistent with primary analysis |
| Calendar-period adjusted model | ICU discharge | Cox model additionally adjusted for pandemic period | Adjusted HR = 2.01 (95% CI, 1.35–2.99) | <0.001 | Consistent with primary analysis |
| APP initiation exposure model | Endotracheal intubation | Exposure defined as first documented APP initiation rather than cumulative APP duration ≥10 h | Adjusted OR = 0.31 (95% CI, 0.13–0.74) | 0.008 | Directionally consistent; attenuated |
| APP initiation exposure model | 28-day all-cause mortality | Exposure defined as first documented APP initiation rather than cumulative APP duration ≥10 h | Adjusted OR = 0.42 (95% CI, 0.17–0.98) | 0.046 | Directionally consistent; attenuated |
| 24-hour landmark analysis | Endotracheal intubation after landmark | Patients intubated or dead before 24 h excluded; exposure defined by APP initiation by 24 h | Adjusted OR = 0.34 (95% CI, 0.14–0.82) | 0.016 | Directionally consistent; attenuated |
| 24-hour landmark analysis | 28-day all-cause mortality after landmark | Patients intubated or dead before 24 h excluded; exposure defined by APP initiation by 24 h | Adjusted OR = 0.45 (95% CI, 0.20–0.99) | 0.048 | Directionally consistent; attenuated |
| 48-hour landmark analysis | Endotracheal intubation after landmark | Patients intubated or dead before 48 h excluded; exposure defined by APP initiation by 48 h | Adjusted OR = 0.37 (95% CI, 0.15–0.90) | 0.028 | Directionally consistent; attenuated |
| 48-hour landmark analysis | 28-day all-cause mortality after landmark | Patients intubated or dead before 48 h excluded; exposure defined by APP initiation by 48 h | Adjusted OR = 0.49 (95% CI, 0.22–1.08) | 0.071 | Directionally consistent; not statistically significant |
| Complete FiO₂ documentation sensitivity analysis | PaO₂/FiO₂ ratio trajectory | Restricted to ABG observations with documented device-set FiO₂ | Group × time F = 24.8 | <0.001 | Consistent with primary oxygenation trajectory analysis |
| Complete FiO₂ documentation sensitivity analysis | PaO₂/FiO₂ ratio at 168 h | Restricted to ABG observations with documented device-set FiO₂ | 266 ± 51 vs. 203 ± 50 mmHg | <0.001 | Consistent with primary analysis |
| Fine-Gray competing-risk analysis | ICU discharge | Death before ICU discharge treated as competing event | Subdistribution HR = 1.82 (95% CI, 1.23–2.69) | 0.003 | Consistent with primary Cox model; attenuated |

Notes: Effect estimates are shown for APP versus control unless otherwise specified. Logistic regression models were adjusted for age, SOFA score, ICI class, peak methylprednisolone-equivalent glucocorticoid dose, baseline PaO₂/FiO₂ ratio ≤200 mmHg, respiratory support mode, and vasopressor dependence. Calendar-period adjusted models additionally included pandemic period as a three-level covariate: pre-pandemic, pandemic, and post-pandemic. Cox and Fine-Gray models used ICU discharge as the event of interest. In the Fine-Gray model, death before ICU discharge was treated as the competing event. APP, awake prone positioning; CI, confidence interval; HR, hazard ratio; OR, odds ratio; PaO₂/FiO₂, arterial oxygen partial pressure to fraction of inspired oxygen ratio; SOFA, Sequential Organ Failure Assessment.

**Supplementary Table S2.** Timing of APP initiation and early outcome events before landmark points

| **Variable** | **Overall matched cohort (n = 120)** | **APP group (n = 60)** | **Control group (n = 60)** |
| --- | --- | --- | --- |
| Time from ICU admission to first APP session, hours | — | 13 (8–21) | Not applicable |
| APP initiated within 24 h after ICU admission | — | 46/60 (76.7%) | Not applicable |
| APP initiated within 48 h after ICU admission | — | 56/60 (93.3%) | Not applicable |
| Intubation before 24-hour landmark | 6/120 (5.0%) | 1/60 (1.7%) | 5/60 (8.3%) |
| Death before 24-hour landmark | 1/120 (0.8%) | 0/60 (0.0%) | 1/60 (1.7%) |
| Patients excluded from 24-hour landmark analysis | 7/120 (5.8%) | 1/60 (1.7%) | 6/60 (10.0%) |
| Patients included in 24-hour landmark analysis | 113/120 (94.2%) | 59/60 (98.3%) | 54/60 (90.0%) |
| Intubation before 48-hour landmark | 12/120 (10.0%) | 3/60 (5.0%) | 9/60 (15.0%) |
| Death before 48-hour landmark | 3/120 (2.5%) | 1/60 (1.7%) | 2/60 (3.3%) |
| Patients excluded from 48-hour landmark analysis | 15/120 (12.5%) | 4/60 (6.7%) | 11/60 (18.3%) |
| Patients included in 48-hour landmark analysis | 105/120 (87.5%) | 56/60 (93.3%) | 49/60 (81.7%) |
| Patients with documented APP initiation but intubation or death before accumulating 10 h of APP exposure before final PSM cohort assembly | 5 | 5 | Not applicable |

Notes: Data are presented as median (interquartile range) or n/total (%). APP initiation was defined as the start time of the first documented APP session in ICU nursing flow sheets or respiratory therapy records. The 24-hour and 48-hour landmark analyses excluded patients who were intubated or died before the corresponding landmark time point. The final row refers to patients identified in the eligible pre-matching source cohort who had APP initiated but did not meet the primary cumulative APP exposure definition because intubation or death occurred before 10 hours of documented APP exposure could be accumulated. APP, awake prone positioning; ICU, intensive care unit; PSM, propensity score matching.
